# Successful intravenous thrombolysis for ischemic stroke after reversal of dabigatran anticoagulation with idarucizumab: a case report

**DOI:** 10.1186/s13256-017-1404-2

**Published:** 2017-08-15

**Authors:** Sergio Agosti, Laura Casalino, Enrico Rocci, Gabriele Zaccone, Eugenia Rota

**Affiliations:** 1 Cardiology Department, San Giacomo Hospital, Novi Ligure, Alessandria Italy; 2 Cardiology Unit, ASL 3, Genoa, Italy; 3 Neurology Unit, San Giacomo Hospital, Novi Ligure, Alessandria Italy

**Keywords:** Dabigatran, Idarucizumab, Ischemic stroke, Non-vitamin K antagonist oral anticoagulants, Thrombolysis

## Abstract

**Background:**

Non-vitamin K antagonist oral anticoagulants, including dabigatran, are currently widely used for the prevention of stroke and systemic embolism in patients with non-valvular atrial fibrillation. Recently, idarucizumab, a monoclonal antibody fragment for immediate reversal of dabigatran-induced anticoagulation, has been introduced into the market to be used in life-threatening bleeding or urgent surgery, allowing for rapid normalization of clotting parameters. The use of idarucizumab is not yet well established in patients presenting with acute ischemic stroke on dabigatran who are candidates for thrombolytic therapy.

**Case presentation:**

We report the case of a 71-year-old hypertensive Caucasian woman with non-valvular atrial fibrillation treated with dabigatran 150 mg twice daily, who presented with acute ischemic stroke causing right-sided hemiparesis and aphasia. Two hours after presentation to the emergency department, a decision was made to administer idarucizumab for achieving complete reversal of any potential anticoagulant effect of dabigatran and, in the absence of any contraindications, our patient underwent successful thrombolysis. At discharge, our patient was able to walk unassisted and had only residual aphasia. Twenty days later, she had completely recovered motor function of her right side, with further progressive improvement of aphasia. Repeat cranial computed tomography confirmed the absence of hemorrhage, and anticoagulant therapy with dabigatran 150 mg twice daily was resumed.

**Conclusions:**

Our case report adds to the evidence that idarucizumab administration is safe in the setting of patients with atrial fibrillation treated with dabigatran who develop acute ischemic stroke requiring thrombolysis.

## Background

Non-vitamin K antagonist oral anticoagulants (NOACs) are currently widely used for the prevention of stroke and systemic embolism in patients with non-valvular atrial fibrillation (AF) [1, 2], and have been shown to have a more favorable efficacy and safety profile than warfarin [3]. Recently, idarucizumab, a monoclonal antibody fragment for immediate reversal of dabigatran-induced anticoagulation, has been introduced into the market to be used in life-threatening bleeding or urgent surgery, allowing for rapid normalization of clotting parameters [4, 5]. However, use of idarucizumab has not yet been well established in patients presenting with acute ischemic stroke on dabigatran who are candidates for thrombolytic therapy [6]. Indeed, case reports addressing this issue are sparse in the literature [7–10]; therefore, prospective studies are warranted to elucidate the safety and efficacy of idarucizumab in this setting.

The following case report details the use of idarucizumab in a patient who presented with cerebral ischemia while undergoing treatment with dabigatran, and who was a candidate for thrombolytic therapy. We show here that idarucizumab was effective and safe for the immediate reversal of dabigatran-induced anticoagulation, and that there were no pharmacodynamic interactions with the thrombolytic therapy.

## Case presentation

A 71-year-old obese Caucasian woman (100 kg, body mass index 33 kg/m^2^) presented to the emergency department at 08:30 p.m. with motor aphasia, ideomotor apraxia, and right facio-brachio-crural hemiparesis that had occurred 1 hour before admission; her National Institutes of Health Stroke Scale (NIHSS) score was 9. She had no other neurological symptoms.

Our patient was fully self-sufficient and retired; she had previously worked as a secretary. She had a history of hypertension dating back to around 15 years and she was on antihypertensive therapy with candesartan (16 mg once daily) and furosemide (25 mg on alternate days). She suffered from paroxysmal AF diagnosed in 2014, and, at presentation, was on sotalol 80 mg three times daily. In November 2015, she was put on oral anticoagulant therapy with warfarin, replaced by dabigatran 150 twice daily in October 2016. Her medical history included thyroid disease in 1987, prior bilateral total hip replacement surgery in 2013, an episode of pulmonary embolism in November 2015, and mild obstructive sleep apnea syndrome.

On admission, her blood pressure was 130/80 mmHg and oxygen saturation (SaO_2_) was 98%. An electrocardiogram (ECG) revealed a normal sinus rhythm with a heart rate of 55 bpm. One week previously, our patient had undergone a cardiology evaluation, and electrical cardioversion of persistent AF was planned.

Urgent brain imaging with computed tomography (CT) did not reveal any ischemic lesions.

Initial blood testing was unremarkable, with a hemoglobin level of 14.4 g/dL (normal range: 12–16 g/dL), and normal renal function with a creatinine level 0.79 mg/dL (0.51–0.95 mg/dL) and a creatinine clearance of 103 mL/min. Her cardiac troponin level was 0.01 ng/mL (0.00–0.4 ng/L) and her coagulation panel revealed an activated partial thromboplastin time (aPTT) of 29 seconds (20–29.6 seconds) with an international normalized ratio (INR) of 1.31 (0.8–1.30).

After family and personal history-taking, it was uncertain whether our patient had been compliant with her prescribed dose of dabigatran.

At 11:00 p.m., a decision was made to administer intravenous idarucizumab (2 × 2.5 g/50 mL) to achieve complete reversal of any potential anticoagulant effect of dabigatran. In the absence of contraindications, our patient received thrombolytic therapy with tissue plasminogen activator intravenously at 0.9 mg/kg body weight according to standard protocol (total dose 90 mg infused over 60 minutes, with 10% of the total dose administered as an initial bolus over 1 minute).

Our patient improved rapidly after thrombolysis and had only minor right hemiparesis with mild improvement of aphasic symptoms within a few days.

**Fig. 1:**
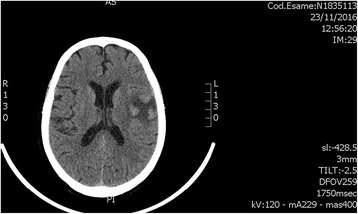
Ischemic hypodensity involving the left Sylvian fissure on brain computed tomography

Serial brain CT scans performed in the following days showed ischemic hypodensity involving the left Sylvian fissure (Fig. 1).

During hospitalization, our patient had an episode of atrial tachyarrhythmia appropriately managed with amiodarone intravenously and oral bisoprolol. She was placed on combination therapy with aspirin (100 mg) and low-molecular-weight heparin (4000 IU twice daily). Doppler imaging of the supra-aortic trunks and echocardiography studies did not show any abnormalities.

At discharge, our patient was able to walk unassisted and had only residual aphasia and a regular ventricular response to AF. She was prescribed aspirin (100 mg daily), bisoprolol (2.5 mg twice daily), ranitidine (150 mg daily), and low-molecular-weight heparin (4000 IU twice daily).

Twenty days after discharge, our patient had completely recovered motor function of her right side, with further progressive improvement of aphasia. A repeat cranial CT scan confirmed the absence of hemorrhage, and anticoagulant therapy with dabigatran 150 mg twice daily was resumed. One week later, her dabigatran plasma levels obtained 2 hours after ingestion were 294 ng/mL (normal range >30 ng/mL) with an aPTT of 47 seconds.

At 6 months follow-up, our patient had complete functional motor recovery, with persistent but mild expressive aphasia; she was still on therapy with dabigatran 150 mg twice daily.

## Discussion

Evidence derived from numerous clinical trials has consistently demonstrated a good safety profile for NOACs, with a relative risk reduction in intracranial hemorrhage of up to 60% [11], even though the management of NOAC-related bleeding still remains a significant challenge in clinical practice [12]. The recent approval of idarucizumab, a monoclonal antibody fragment specifically targeted to reverse the direct thrombin inhibitor dabigatran, has provided the medical community with an important tool for the management of patients with uncontrolled bleeding.

The REVERSE-AD study enrolled patients treated with dabigatran who presented to the emergency department with life-threatening bleeding, or who required urgent surgery or intervention. Idarucizumab completely reversed the anticoagulant effect of dabigatran within minutes in both patients groups, suggesting that this antidote is safe and effective [4].

Several case reports also demonstrated the efficacy of idarucizumab in specific clinical settings, including patients with acute ischemic stroke eligible for thrombolysis [13]. Intravenous thrombolytic therapy is contraindicated in anticoagulated patients, and the availability of an effective means to ensure rapid restoration of hemostasis seems to further improve the chances of a successful recanalization of occluded vessels in the acute phase of an ischemic stroke [14, 15].

Our patient could not remember whether she had been compliant with her prescribed dose of dabigatran. While values of aPTT in the normal range appeared to confirm the possibility of noncompliance, further investigations (for example, counting remaining pills or questioning family members) were not undertaken because of the narrow time window for intravenous thrombolysis. However, aPTT provides only a qualitative measure of the anticoagulation status and does not have a linear relationship with dabigatran plasma concentration. Therefore, administration of idarucizumab prior to thrombolysis was deemed opportune [16].

Because of NOACs’ short half-life (approximately 12 hours), even one missed dose may lead to subtherapeutic anticoagulation, exposing patients to an increased risk of systemic thromboembolism and intracranial hemorrhage. Therefore, compliance and adherence to treatment is crucial. We assumed that our patient had been noncompliant with her prescribed dose of dabigatran, which may well account for the occurrence of ischemic stroke, and we decided to resume dabigatran administration after 3 weeks in the absence of any findings suggestive of lack of efficacy.

Seven days after resumption of therapy, assessment of dabigatran plasma concentrations and routine coagulation parameters confirmed an effective return of anticoagulant activity following regular intake of the medication and, therefore, the therapy could be continued safely.

Compared with other published cases, this clinical case report is relevant in that idarucizumab was given under conditions in which dabigatran was only partially effective, and therefore had a less-than-optimal anticoagulant profile. Despite this, idarucizumab did not show a pro-thrombotic effect, and there were no pharmacodynamic interactions with the thrombolytic therapy.

## Conclusions

In situations of life-threatening bleeding or urgent surgery, the immediate reversal of dabigatran-induced anticoagulation by idarucizumab can rapidly normalize clotting parameters and avoid morbidity. Our case report adds to the evidence that idarucizumab administration is safe in the setting of AF patients treated with dabigatran who develop acute ischemic stroke requiring thrombolysis, although further prospective studies are warranted to define the optimal management strategy for these patients [17].

## Abbreviations

AF: Atrial fibrillation
aPTT: Activated partial thromboplastin time
CT: Computed tomography
ECG: Electrocardiogram
INR: International normalized ratio
NIHSS: National Institutes of Health Stroke Scale
NOACs: Non-vitamin K antagonist oral anticoagulants
SaO_2_: Oxygen saturation
